# Green human resource management practices to accomplish green competitive advantage: A moderated mediation model

**DOI:** 10.1016/j.heliyon.2023.e21830

**Published:** 2023-10-31

**Authors:** Khurram Mustafa, Md Billal Hossain, Farooq Ahmad, Faisal Ejaz, Hafiz Ghufran Ali Khan, Anna Dunay

**Affiliations:** a MS Scholar in Management, Faculty of Management Sciences, University of Okara, Okara, Pakistan; b Business Management and Marketing Department, School of Business and Economics, Westminster International University in Tashkent (WIUT), Tashkent 100047, Uzbekistan; c Department of Business Administration, Fatima Jinnah Women University, Rawalpindi, Pakistan; d School of International Relations, Minhaj University, Lahore, Pakistan; e Faculty of Management Sciences, International Islamic University, Islamabad, Pakistan; f John von Neumann University, Doctoral School of Management and Business Administration, 1117 Budapest, 6000 Kecskemét, Hungary

**Keywords:** Green human resource management practices, Green knowledge sharing, Green innovation, Green competitive advantage, Green human capital

## Abstract

In present high-tech era, firms need to possess a variety of capabilities and resources to attain and sustain a competitive position in the market. The motivation for this study was to understand green competitive advantage through the application of Ability-Motivation-Opportunity theory and Natural-Resource-Based view. In a time-lagging longitudinal online survey related to small-and-medium-sized manufacturing enterprises, 223 professionals provided data according to their opinions. The structural and measurement model were designed for analyses. The results supported the model and verify the Green human resource management practices’ influence on green competitive advantage, with partial mediation of green knowledge sharing and green innovation (green product innovation and green process innovation). The analyses revealed the positive highly significant moderation of green human capital, which is the novelty of the study. Green human capital is important to develop sustainable workforce who act as catalyst in achieving sustainable development goals. The report offers practical advice for small-medium manufacturing enterprises (SMMEs) aiming to attain a green competitive advantage. With the help of a green competitive advantage, the recommendations in this study can benefit SMMEs to develop a green human capital and to create innovative knowledge. As a result, it is a futuristic approach to dealing with the improved environmental conditions and developed a green human capital in this industrialized age.

## Highlights

• Green HRM practices promote green competitive advantage (GCA).
• Green knowledge sharing (GKS) mediates between Green HRM and Green innovation.
• Green innovation (product and process) mediates between GKS and GCA.
• GKS and Green innovation act as serial mediators between Green HRM and GCA.
• Green human capital strengthens the link between Green HRM practices and GKS.

## 1 Introduction

Sustainability is the millennial strategic goal of the modern global organizations [1,2]. The Sustainable Development Goals and targets are unified and indivisible, worldwide in scope and all around relevant, thinking about different nations' real factors, limits, and levels of advancement, as well as public approaches and destinations [3]. The impact of green aspects of firms shows up more straightforward and notable [4], furthermore naturally horrendous cases, for example, air or water contamination and atomic power mishaps have reignited worries about the adverse consequences of industrialization. Hence, environmental management is a tool for long-term sustainability, particularly for SMMEs [5]. This study concentrated on Pakistan's Punjab province SMMEs, which are the industry's backbone.

Strategically oriented Human Resource Management (HRM) can help a business achieve its goals for corporate social responsibility [6]. The growth of this function has prompted related business initiatives and research, labeled Green HRM practices. Since the last decade, this field has grown rapidly [6]. This terminology was created because management realized that inefficient use of resources by human resources was contributing to organizational impact on the environment [7] as well as the triple bottom-line [8]. SMMEs in Pakistan has the ability to enable environmental improvements, benefit green employee behavior, and a positive green image in the market [[9], [10], [11], [12]].

Green HRM practices include green recruitment and selection, green performance management, green training and development, green employee involvements, and green pay and rewards [13]. Because high involvement of management leads to corporate profitability as well as employee's need satisfaction at workplace [14]. This behavior leads to an opportunity like green knowledge sharing (GKS) [15,16]. Hence, there is a gap existed in the literature that how these Green HRM practices impact GKS, if skilled labor in the form of green human capital (GHC) has already strong impact on the organization to get sustainable performance [17]. Therefore, this study answered this question by using quantitative approach to generate fresh literature and to highlight the novelty of GHC in this relationship. Because, the role of green growth in reducing carbon dioxide (CO_2_) emissions has been empirically and theoretically examined in the previous literature, particularly when GHC is considered a crucial factor in a sustainable environment [18].

To get a green competitive advantage (GCA) in environmentally-friendly products, organizations should yield products that have both greenness and breakthrough features, according to this study. This segment of GCA is based on natural-resource-based view (NRBV) framework. Furthermore, there is a gap exists in the literature that how green process innovation (GPSI) is providing capabilities to the organization in order to achieve GCA [17,19]. This study provides knowledge to fill the gap as well as throws light on its mediator role with GKS [20], in the context of SMMEs in the Punjab province of Pakistan. The following research questions must be addressed in further detail for business perspective.

• In achieving the GCA, what are the organizational consequences regarding motivations of Green HRM practices?
• How did Green HRM practices motivate employees to gain opportunity like GKS, which resulted to green innovation (GI) and ultimately helped the organization to realize a GCA?
• How much moderation of GHC is present in the connection of GKS and Green HRM practices?
• What level of GKS and GI mediates the relationship of Green HRM practices and GCA?

The background and hypotheses statements about the variables are elaborated in the upcoming section. Furthermore, the methodology section identifies the sampling approaches to collect the data from the SMMEs. Then, results and analysis presents the validity and reliability of measures and study results computed by using various software packages. After all, the discussion section explains the contributions of the study as well as the future research directions.

## 2 Theoretical background and hypotheses development

### 2.1 Theoretical underpinning

**Fig. 1:**
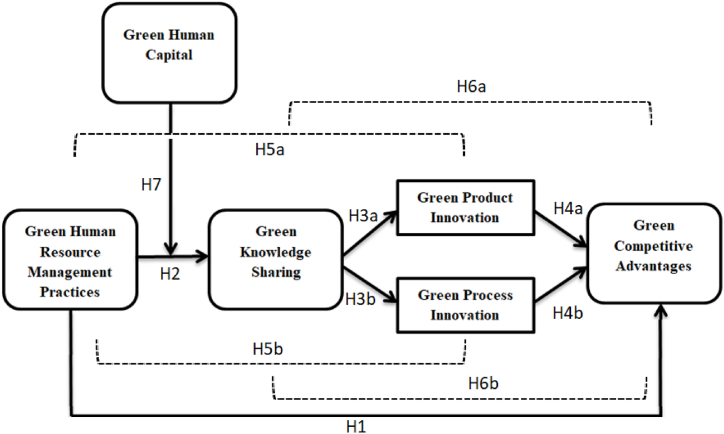
Theoretical model.

The theoretical framework for this study is shown in Fig. 1. The first chunk of the theoretical model used ability-motivation-opportunity (AMO) theory. It explains the Green HRM practices in the most dominant fashion and identifies as high performance work practices [21,22]. Hence, these practices are based on three main aspects named ability, motivation and opportunity [21]. Firstly, ability practices explain about the require knowledge and skills to perform tasks. This study named GHC as the ability practices. Secondly, motivation practices provide financial and non-financial incentives to the employees in order to enhance the efforts of to achieve the assigned targets [23]. Although, incentives are the portion of Green HRM practices therefore, this study has chosen it as the motivation aspect of the theory. At last, opportunity practices enhance employee participation by providing more autonomy to the employees such as GKS and involvement [24]. This study selected GKS as opportunity practices. Therefore, AMO theory explains variables like GHC as ability practices, Green HRM practices as motivation practices and GKS as opportunity practices in the theoretical model of this study.

The second fragment of the research model is NRBV. Based on the capabilities that support environment-friendly economic activities, it is a cutting-edge strategy and competitive advantage [7]. This theory uses three types of capabilities that provide a way out to gain GCA i.e., pollution prevention, product stewardship and sustainable development. Firstly, pollution prevention reduces, changes or prevented from the emissions and effluents through continuous improvement mindset, material substitution, recycling or process innovation to protect the environment [7]. This study used GPSI as variable in the framework from the pollution prevention capability.

Secondly, product stewardship is the capability that minimize the life-cycle costs from the design or development process of the product through stakeholder integration in order to preempt competitors [7]. This is also termed as product innovation and lead to the GCA. This study used green product innovation (GPTI) as variable from product stewardship capability. Whereas, the sustainable development capability minimizes the environmental burden of organizational growth and development from the economic activities.

### 2.2 Study variables

#### 2.2.1 Green HRM practices

It is a term described as those aspects of sustainable HRM that deal with environmental sustainability concerns [25]. Similarly, these are the activities that positively affect the environmental consequences [26]. Moreover, Green HRM practices consider green behaviors in performance appraisals, rewards, compensation and promotion to motivate employees about environment-friendly activities [27]. Therefore, this study designated Green HRM practices as independent variable and motivation factor according to AMO theory. Furthermore, these practices may be improved by providing green training and development to the employees or by setting green goals for employees. Hence, Green HRM practices are the green management aspects of HRM [27,28].

#### 2.2.2 Green knowledge sharing

A process in which employees share environment-friendly implicit and explicit information to produce new knowledge is called GKS within a firm [29]. For example, collaborating with colleagues to share environmental knowledge and skills that are advantageous to the employees and organization [30]. The fact that implicit knowledge exists in the human brain makes it challenging to codify. Contrarily, explicit knowledge is easily demonstrated because it is recorded in formal, official documents [16,31]. Furthermore, AMO theory along with this study considers GKS as mediating variable and opportunity factor in order autonomize the employee participation towards green goals [23]. Moreover, it is less focused by researchers that effect the organization [15,16]. Therefore, by advancing this idea, this study focused on this gap in the literature.

#### 2.2.3 Green product innovation

As segment of product stewardship, it aims to modify or adapt product designs to lessen their negative effects on the environment [32]. Furthermore, it includes This type of innovation is used as a weapon to attain GCA in the market. Such as, organization may choose the raw material that produces less pollution and removal of hazardous substances from the product [33,34]. Moreover, it is also the portion of NRBV's product stewardship capability which provide organizations the first mover advantage [7]. Hence, this study used it as mediator and predictor of GCA.

#### 2.2.4 Green process innovation

It reduces the emissions, effluents or waste or makes it a valuable resource during the production process [7,34]. For example, lowering energy and resource use, cutting back on water use, increasing resource efficiency, and switching to bioenergy from fossil fuels [33]. Therefore, GPSI helps organization to take GCA in the form of cost efficiency and profitability [35]. Furthermore, NRBV consider it as the capability of pollution prevention from the environment [7] and this study consider it as mediator and predictor of GCA.

#### 2.2.5 Green competitive advantage

It is a situation in which competitors could not copy a certain position of an organization regarding environment protection or GI [36]. Such as, it may in the form of low cost about GI, better quality or profitability of green products or services, capability of research and development about environmental protection, managerial capabilities and first mover advantage [36]. According to NRBV, organization gets sustainable benefits from these strategies in the form of creating new markets and improving green organizational image [7,[37], [38], [39]]. This concept has very limited knowledge available in the literature. Therefore, this study highlighted its importance and used it as dependent variable in the framework.

#### 2.2.6 Green human capital

Employees knowledge, skills, experience, innovation, wisdom, attitude, commitments and creativities in the aggregation, to adopt environmental protection policies and procedures, is known as GHC [[40], [41], [42]]. Examples include competencies, contributions and productivity, green product or service quality, team work cooperation and management support in order to achieve environmental protection goals [40]. Moreover, it has noteworthy in adapting Green HRM practices and is the individualistic ability in the organizational science [43,44]. Hence, this study contemplates GHC as ability factor according to AMO theory and reflects as moderating variable in the theoretical framework.

### 2.3 Hypothesis development

#### 2.3.1 Green HRM practices and green competitive advantage

The most prevalent notion is the AMO theory in understanding the impact of Green HRM practices in the form of high-performance work practices on organizational performance like a GCA in empirical studies [21,22]. The methods which guarantees that workers have the knowledge and skills required to do certain activities. Such as recruitment and selection with the training and development programs, create the foundation of how successfully people are able to accomplish their jobs. Similar to this, motivation is based on procedures like performance appraisals with monetary and non-monetary rewards that are intended to motivate workers to put in more effort in order to fulfill performance objectives. This will help the organizations to move strategically as well as employee well-being may be considered [45]. Finally, opportunity is a combination of behaviors that combines participation, knowledge sharing, and actions that boost autonomy to encourage employee participation in activities [24]. Hence, Green HRM practices are based on AMO theory because these practices are identified as high-performance work practices.

According to NRBV, a sustained competitive advantage may be achieved through pollution prevention, sustainable development and product stewardship [7]. Whereas, sustainable development is the process for shared vision of a firm-specific resource, which is rare in terms of competitors. All of three processes adopted by a firm may help them to achieve GCA. Therefore, the two frameworks have collaborated in this study i.e. AMO framework and NRBV framework. Green competitive advantage needs employee involvement for pollution prevention and further sustainable developments. The employee involvement is the product of Green HRM practices which may be achieved through compensation and rewards on performances. As a result, Green HRM practices from AMO framework is combined to achieve the GCA from NRBV framework, which is a desired outcome for a firm. Hence, this discussion concludes that.

H1 Green HRM practices positively influence the organization's GCA.

#### 2.3.2 Green HRM practices and green knowledge sharing

In the pro-environmental agenda, self-perceptions in the form of skill, inspiration, prospect and knowledge is necessary to practice green behaviors when AMO framework taking into play [46]. Therefore, a relationship exists between Green HRM practices and GKS in the AMO framework. For businesses to retain a long-term competitive advantage, knowledge and knowledge dissemination are deemed critical [47]. The strong impact of GKS also leads to employee environmental commitment in order to improve environmental performance [48,49]. Through successful development strategies, relational connections, criticism, support, and creating information stocks, Green 10.13039/100005448HRM practices advance towards information transfer and creation as well as individual learning among employees [50]. Accordingly, this exploration trusts that assuming employees have an ideal demeanor toward Green HRM practices; they will be bound to share eco-friendly information. Therefore.

H2 Green HRM practices positively influence the organization's GKS.

#### 2.3.3 Green knowledge sharing and green innovation

Knowledge absorbing firms create the tentative outcomes available; as a result other organizations may utilize the information for creative reasons, promoting collective innovation, in order to achieve a win-win culture for all [51]. Therefore, organizations need innovative and unique knowledge to create environment-friendly products in order to fulfill customer and stakeholder demands [34,52]. Since successful innovations are closely related to the wider spectrum of knowledge sources, previous literature suggests that organizations should look more broadly for superior research and development results [53,54]. Furthermore, GPTI is a way to reduce the environmental consequences of disposal with enhancing energy efficiency by employing nontoxic chemicals or biodegradable materials throughout the manufacturing process [32]. Hence, life-cycle analysis provides an overall picture to reduce the environmental burden by this system from cradle to grave [7]. Consequently, this study draws a statement that GKS provides an opportunity to the organization in order to develop green products innovation. This will evaluate the relationship between the GPTI from NRBV framework and GKS from AMO framework. So.

H3a GKS positively influences the organization's GPTI.

Green process innovation makes improvement in the production procedures with the aim to reduce the waste, capitalization of renewable resources and cut the resource consumption [55,56]. Social networking, meetings and collaborations provide ways of learning and knowledge sharing which improves organizational operation and finally organizational objectives [57,58]. Similarly according to NRBV framework, pollution prevention minimizes the emission and effluents by using better material substitution, recycling and GPSI [7]. This process removes pollution from the saleable goods of the organization. Therefore, Green HRM practices in AMO framework provides green compensations and rewards to promote GKS as an opportunity. The open innovation mindset of employees consists of beliefs, values and attitudes which captures the employee openness towards knowledge sharing and sourcing within or outside the firm borders [59]. Hence, this study links GKS and GPSI to join the AMO framework with NRBV framework. Therefore.

H3b GKS positively influences the organization's GPSI.

#### 2.3.4 Green innovation dimensions and green competitive advantage

Product stewardship includes the voice of environment into the product design or development processes, according to NRBV framework [7]. Product life-cycle analysis includes the minimization of nonrenewable materials, removal the usage of toxic materials and renewable materials according to their replenishment [60]. Therefore, these green products may help to capture the GCA by competitive preemption [61]. Moreover, two means may help to achieve this GCA. These are limited resources like raw production capacity, raw materials or customers are important to gain as preferred access, and by establishing uniquely tailored standards, rules, regulations to the organization's capability [62]. For example, BMW has gained an early reputation of recycling and taking back its products. This preemption was called design for environment (DfE) automobiles [7] which leads to a GCA. Therefore.

H4a GPTI positively influences the organization's GCA.

Green process innovation rallies the operational performance of the organization in the form of productivity, improved efficiency and reduced waste disposal costs [63,64]. Such as, Toxic release inventory (TRI) has restricted to organizations to manage their emissions to 300 hazardous chemicals or toxics [7]. Thus, these technological capabilities play an important role in sustained competitive advantage and enhancing innovation [64,65]. So, in order to achieve the desired performance outcomes, the development of new technologies is required by GPSI [66].

According to NRBV, organizations have understood now that pollution rose with inefficient use of materials and human resources. Therefore, they move towards the concept of continuous improvement of total quality environmental management [7]. This process required a better material substitution, housekeeping or recycling which is named as GPSI [67]. Moreover, organizations may achieve significant savings in the form of cost advantage relative to competitors, considers as GCA [68]. Hence.

H4b GPSI positively influences the organization's GCA.

#### 2.3.5 Mediation of green knowledge sharing and green innovation dimensions

According to some studies [[69], [70], [71]], the process of developing GI is openly backed by the influence of GKS during production. AMO theory also confirms this concept as Green HRM practices have the opportunity in the form of GKS for organizations [23]. This study will examine that how GKS mediates the relationship between Green HRM practices and GPTI. This research will also exhibit the mediating role of GKS in the relationship of Green HRM practices and GPSI. Therefore.

H5a GKS positively mediates the link between Green HRM practices and the organization's GPTI.

H5b GKS positively mediates the link between Green HRM practices and the organization's GPSI.

Innovation will result from the constant collection and integration of new knowledge [72]. Moreover, the unseen accumulation of experience as well as evident and visible knowledge, contribute to the creativity required for innovation [73]. Hence, product is improved according to environment. Therefore, this process will preempt the organization as compared to competitors. This preemption actually leads to GCA for the organization. Hence.

H6a Organization's GPTI positively mediates the link between GKS and the organization's GCA.

Organizations must actively seek out, create, transform, and apply knowledge in order to develop unique ideas and knowledge with the help of GPSI [64]. Therefore, these organizational strategic and sustainable capabilities improve the operational performance [63]. As a result, this study develops the statement that improvement in operational performance may lead towards GCA. Hence.

H6b Organization's GPSI positively mediates the link between GKS and the organization's GCA.

#### 2.3.6 Moderation of green human capital

Employees are now seen as one of the most significant intangible asset and environmental novel build is the name given to expertise as ‘GHC’ [41,42,74]. Therefore, it falls in the ability category of AMO theory, according to this study. If a well-developed and well-equipped workforce is already available in the organization then this workforce may support better towards newly recruited employees for environmental synergy [75]. This will only happen when they collaborate and disseminate knowledge with one another properly. Though, this study used GHC as moderator role in the framework. Therefore.

H7 GHC moderates the relationship between Green HRM practices and GKS such that, at high levels of GHC, this relationship becomes more robust.

## 3 Methodology

### 3.1 Data collection and sample

Small and Medium Enterprises Development Authority (SMEDA) is an independent government agency under the Ministry of Industries and Production in Pakistan. The study selected business level as the unit of analysis. Thus, the study's scope is confined to 400 SMMEs in Punjab province of Pakistan [76]. Organizations having employees 250 or less are known as SMMEs in Pakistan [77]. So that, the study may able to maximize the variation across numerous work contexts by gathering data from a range of SMMEs. Furthermore, it is necessary for SMMEs to adopt sustainable development in order to achieve GCA [36].

Cross-sectional data collecting has been recognized by researchers as a design flaw and longitudinal designs or time-lagged have been suggested as a solution [[78], [79], [80]]. In this study, a three point time-lagged design was used to collect data of different variables at different time periods. At time 1 (T_1_), information on Green HRM practices and GHC was gathered. Three months later, at time 2 (T_2_), data on GKS, GPTI and GPSI were gathered using an online poll. At time 3 (T_3_), information on GCA was gathered (2 weeks after time 2) [81].

This empirical study adopted simple random sampling technique to collect data. Organizations were picked on the basis of environmental awareness [82] and governed by government rules [83]. Online questionnaire approach was adopted in this study to collect data with the help of WhatsApp and email applications [[84], [85], [86]]. This approach discovered a total of 223 professional responses, grounded on the quantity of employees. Additional investigation confirmed their names, cellphone numbers, email addresses and job titles [36].

The study received 247 responses at T_1_ out of the 400 questionnaires that were initially disseminated via WhatsApp and email. At T_2_, 236 respondents in total returned finished questionnaires three months later. Finally, at T_3_, 2 weeks following T_2_, 223 responses were received, which were included in the final sample, the total response rate was 55.8 %. Responses were gathered in between November 2021 as T_1_, February 2022 as T_2_ and finally March 2022 as T_3_. All responses to the previously listed factors were collected on a 7-point Likert scale, with 1 denoting “strongly disagree” and 7 denoting “strongly agree”. The respondent SMME-sectors included sugar industry (30.9 %), food industry (27.4 %), leather industry (21.1 %), medicine industry (9 %), textile industry (4 %), paper industry (3.6 %) and others (4 % included feed industry, electronics industry and vegetable oil industry).

### 3.2 Measures

The measures were taken from verified and authentic sources. Table 2 lists the liability indicators of variables.

#### 3.2.1 Green HRM practices

Green HRM practices (GHRM) were measured using six-items [27]. The scale (Composite reliability/CR = 0.92, Cronbach's Alpha/α = 0.89, Average variance extracted/AVE = 0.64) indicated good reliability in this study.

#### 3.2.2 Green knowledge sharing

GKS scale that this study adopted is a five-item measure [30]. The scale showed a good level of reliability in this study (CR = 0.88, α = 0.84, AVE = 0.60).

#### 3.2.3 Green innovation

To assess GI an eight-item measure was adopted [39]; four-items for GPTI and four items for GPSI. Both measures of GI indicated good reliability in this study (GPTI CR = 0.88, α = 0.81, AVE = 0.64; GPSI (CR = 0.87, α = 0.81, AVE = 0.64)).

#### 3.2.4 Green competitive advantage

GCA was assessed using eleven items [36]. The scale (CR = 0.94, α = 0.93, AVE = 0.60) indicated good reliability in this study.

#### 3.2.5 Green human capital

GHC was measured using five items [40]. The scale (CR = 0.87, α = 0.82, AVE = 0.57) indicated good reliability in this study.

## 4 Results

Measurement model and structural model are used in the analysis method to check validity and reliability. Furthermore, mediation and moderation analyses are key to understand the results.

### 4.1 Measurement model

**Fig. 2:**
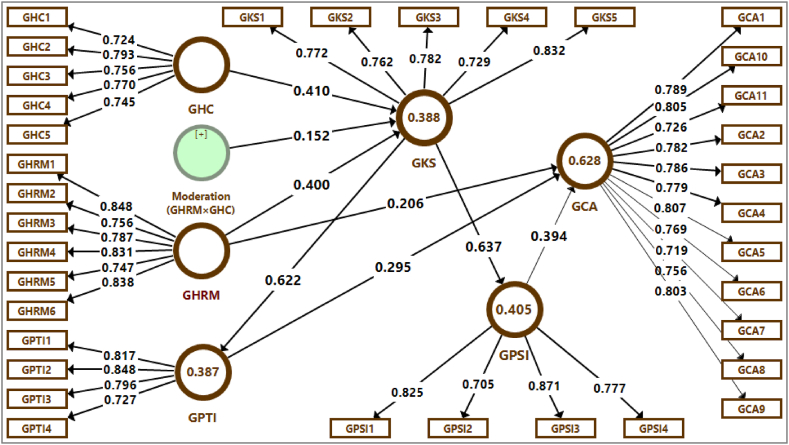
Measurement model.

**Fig. 3:**
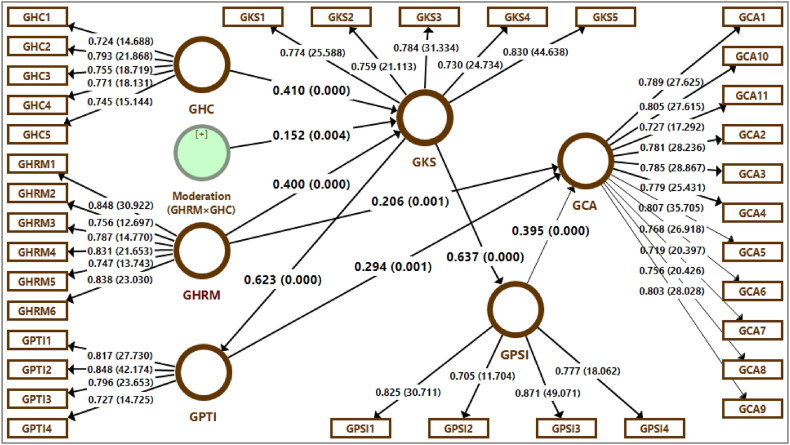
Estimated PLS path model.

The analyses included inter-construct correlations, descriptive statistics and reliability coefficients. Moreover, SPSS version-26 and SmartPLS version-3 were the packages to analyze the model. This investigation pointed out that the measurement model (Fig. 2) has very good convergent validity (Table 1) and discriminant reliability (Table 2). The results showed that these are all above the benchmarked 0.700 in Fig. 3 and Table 1. All other values like composite reliability (>0.700), average variance extracted (AVE) (>0.500) and Cronbach's Alpha (>0.700) were lit the least requirements. All AVE values are lower than the composite reliabilities of their respective constructs. The diagonals of √AVE were greater than its correlations values. It means that results were above the benchmarked values about the discriminant validity of all variables to apply [87]. Furthermore, all R^2^ values (>0.100) were also meeting the required quality criteria for constructs i.e. GKS, GPTI, GPSI and GCA [88].

**Table 1:** Sample Demographic attributes.

Characteristics	Respondents (n = 223)	Percentage (%)
*Gender*		
Male	218	97.8
Female	5	2.2
*Marital Status*		
Married	127	57.0
Unmarried	96	43.0
*Age*		
30 Year or Less	125	56.1
31–40 Year	61	27.4
41–50 Year	34	15.2
Greater than 50 Year	3	1.3
*Educational Qualification*		
Graduation	140	62.8
Masters	80	35.9
Doctoral	3	1.3
*Employee Experience within current Organization*		
1–5 Year	158	70.9
6–10 Year	34	15.2
11–15 Year	14	6.3
More than 15 Year	17	7.6

**Table 2:** Convergent validity.

		Factor Loadings					
Construct/Variable	Items	Minimum	Maximum	AVE	CR	α	R^2^
**GHRM**	6	0.747	0.848	0.643	0.915	0.889	–
**GKS**	5	0.729	0.832	0.602	0.883	0.835	0.388
**GPTI**	4	0.727	0.848	0.637	0.875	0.809	0.387
**GPSI**	4	0.705	0.871	0.635	0.874	0.808	0.405
**GCA**	11	0.719	0.807	0.601	0.943	0.933	0.628
**GHC**	5	0.724	0.793	0.574	0.871	0.815	–

Note: n = 223, α = Cronbach's alpha, AVE = average variance extracted, CR = composite reliability, R^2^=Coefficient of determination, GHRM = Green HRM practices, GKS = Green knowledge sharing, GPTI = Green product innovation, GPSI = Green process innovation, GCA = Green competitive advantage, GHC = Green human capital.

### 4.2 Correlational and descriptive analysis

It included correlation coefficients, mean, standard deviations about constructs in Table 2. Green HRM practices showed substantial positive associations with GKS (*β* = 0.482, *p* < .01), GPTI (*β* = 0.547, *p* < .01), GPSI (*β* = 0.579, *p* < .01) and GCA (*β* = 0.593, *p* < .01), as predicted. The mediator GKS exhibits highly significant and positively correlated with GPTI (*β* = 0.617, *p* < .01) and GPSI (*β* = 0.627, *p* < .01). Both mediators GPTI (*β* = 0.711, p < .01) and GPSI (*β* = 0.739, p < .01) were highly significant and positively correlated with dependent variable GCA. Therefore, all the constructs were positive and highly significant correlations with one another as speculated earlier.

Moreover, all these highly significant correlations of constructs were less than the diagonal values (√AVE) with other constructs and meeting the criteria very well. The moderator GHC also reflected highly significant correlations with other constructs.

Green HRM practices (*β* = 0.538, *p* < .01), GKS (*β* = 0.524, *p* < .01), GPTI (*β* = 0.619, *p* < .01), GPSI (*β* = 0.489, *p* < .01) and GCA (*β* = 0.528, *p* < .01) were highly significant and correlated with it. These correlations showed that theoretical links might be projected in this inquiry without running the risk of multicollinearity.

The heterotrait-monotrait ratio (HTMT) in Table 3 also directed that almost all the required indicator correlations were within the threshold mark (<0.900). Hence, the results exhibited that the discriminant validity was not the issue in the study.

**Table 3:** Correlation and discriminant validity.

	**Mean**	**SD**	**1**	**2**	**3**	**4**	**5**	**6**
**1. GHRM**	4.31	0.560	**0.802**					
**2. GKS**	4.17	0.568	0.482**	**0.776**				
**3. GPTI**	4.03	0.761	0.547**	0.617**	**0.798**			
**4. GPSI**	4.01	0.814	0.579**	0.627**	0.777**	**0.797**		
**5. GCA**	4.09	0.625	0.593**	0.666**	0.711**	0.739**	**0.775**	
**6. GHC**	4.07	0.663	0.538**	0.524**	0.619**	0.489**	0.528**	**0.758**

Note: n = 223, **p < .01, SD = standard deviation, boldface values in the diagonal are √AVE, GHRM = Green HRM practices, GKS = Green knowledge sharing, GPTI = Green product innovation, GPSI = Green process innovation, GCA = Green competitive advantage, GHC = Green human capital.

### 4.3 Structural model and hypothesis Testing

Hypotheses were evaluated in Table 4, Table 5 with the help of direct, specific indirect and total effects with applying partial least square (PLS) approach. This study started by examining the cumulative and direct effects of Green HRM practices, GCA, and GKS. Second, GPTI and GPSI's direct and cumulative effects of GKS were assessed. As well as the effects of GPTI and GPSI on GCA were studied. In order to observe the mediation effect of GKS, GPTI, and GPSI on the relationship between GHRM and GCA, the four-step technique was adopted [89].

**Table 4:** Heterotrait-monotrait ratio (HTMT).

	**GHRM**	**GKS**	**GPTI**	**GPSI**	**GCA**	**GHC**
**GHRM**						
**GKS**	0.562					
**GPTI**	0.646	0.751				
**GPSI**	0.682	0.755	0.959			
**GCA**	0.652	0.757	0.818	0.849		
**GHC**	0.635	0.635	0.762	0.600	0.610	

Note: GHRM = Green HRM practices, GKS = Green knowledge sharing, GPTI = Green product innovation, GPSI = Green process innovation, GCA = Green competitive advantage, GHC = Green human capital.

**Table 5:** Path coefficients (direct and specific indirect effects).

	***β***	**SD**	***t*- Statistics**	***p*-Values**
**GHRM → GCA**	0.206**	0.063	3.262	0.001
**GHRM → GKS**	0.400***	0.099	4.021	0.000
**GKS → GPTI**	0.623***	0.045	13.764	0.000
**GKS → GPSI**	0.637***	0.035	18.058	0.000
**GPTI → GCA**	0.294**	0.085	3.452	0.001
**GPSI → GCA**	0.395***	0.087	4.524	0.000
**GHRM → GKS → GPTI**	0.249***	0.065	3.814	0.000
**GHRM → GKS → GPSI**	0.255***	0.067	3.829	0.000
**GKS → GPTI → GCA**	0.183**	0.055	3.325	0.001
**GKS → GPSI → GCA**	0.252***	0.059	4.231	0.000
**Moderation (GHRM×GHC) → GKS**	0.152**	0.053	2.873	0.004

Note: n = 223, SD = standard deviation, **p < .01, ***p < .001, GHRM = Green HRM practices, GKS = Green knowledge sharing, GPTI = Green product innovation, GPSI = Green process innovation, GCA = Green competitive advantage, GHC = Green human capital..

The investigation also looked at how (GHRM × GHC) affected GKS in a moderating way. The path coefficients of hypotheses indicated that the relationships were highly significant and positive. As a consequence, these findings supported all of the study's hypotheses. The *t*-statistics (>1.96) of direct effects and indirect effects were also highly significant as their values were above the benchmark value. If the analyses check the *p*-values of the relationships then the results came to know that almost all relationships were highly significant and positive.

The positive and high significance were indicated in all the direct and indirect effects of the relationships because these values were within the benchmark limit (*p* < .01). The positive and high significance were indicated in all the direct and indirect effects of the relationships because these values were within the benchmark limit (*p* < .01).

**Fig. 4:**
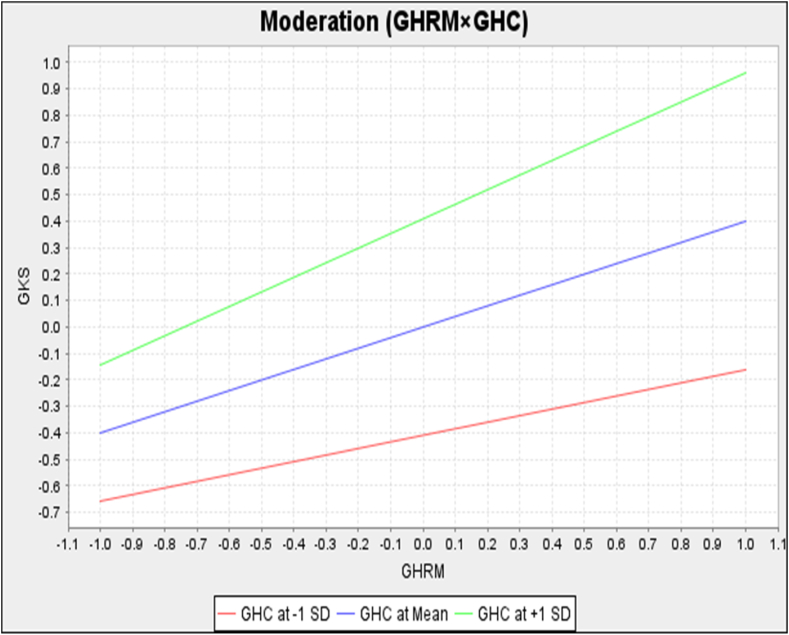
Simple slope analysis of moderation.

The moderating relationship exhibited that there was highly significant effect (*p* < .01) of (GHRM × GHC**→**GKS). The moderating effect (GHRM × GHC**→**GKS) was positive and highly significant which was determined by (*β* = 0.152, *t* = 2.873, *p* < .01) as well. The estimated PLS path model and the complete moderated-mediation model (Fig. 4) were determined. It shows that GHRM (*β* = 0.400) and GHC (*β* = 0.410), and their interaction term (GHRM × GHC; *β* = 0.152) explain a 38.8 % variance in GKS (R^2^ = 0.388). According to Hypothesis 1, GHRM has a substantial positive relationship with GCA.

The direct impact of GHRM on GCA with mediation is positive and highly significant (*β* = 0.206, t = 3.262, p.01), as are the overall effects without mediation (*β* = 0.380, t = 4.968, p.01), verifying Hypothesis 1. Moreover, Hypothesis 2 states that GHRM and GKS have a strong positive relationship. The direct and total effects of GHRM on GKS (*β* = 0.400, t = 4.021, p < .01) are positive and highly significant. GKS shows a substantial positive correlation with GPTI, according to Hypothesis 3a. This hypothesis supported by the fact that the overall impact of GKS on GPTI (*β* = 0.623, *t* = 13.764, *p* < .01) was positive and highly significant (see Table 6). GKS shows a substantial positive correlation with GPSI as well, according to Hypothesis 3b. The hypothesis 3b is supported by the fact that the impact of GKS on GPSI (*β* = 0.637, *t* = 18.058, *p* < .01) was also positive and highly significant.

**Table 6:** Path analysis and hypotheses Testing.

**Path**	**Overall Impact (*t*- Statistics)**	***p*-Values**	**Hypothesis**	**Outcome**
**GHRM → GCA**	0.380*** (4.968)	0.000	H_1_	Supported
**GHRM → GKS**	0.400*** (4.021)	0.000	H_2_	Supported
**GKS → GPTI**	0.623*** (13.764)	0.000	H_3a_	Supported
**GKS → GPSI**	0.637*** (18.058)	0.000	H_3b_	Supported
**GPTI → GCA**	0.294** (3.452)	0.001	H_4a_	Supported
**GPSI → GCA**	0.395*** (4.524)	0.000	H_4b_	Supported
**GHRM → GKS → GPTI**	0.249*** (3.814)	0.000	H_5a_	Supported
**GHRM → GKS → GPSI**	0.255*** (3.829)	0.000	H_5b_	Supported
**GKS → GPTI → GCA**	0.183** (3.325)	0.001	H_6a_	Supported
**GKS → GPSI → GCA**	0.252*** (4.231)	0.000	H_6b_	Supported
**(GHRM** × **GHC) → GKS**	0.152** (2.873)	0.004	H_7_	Supported

Note: n = 223, **p < .01, ***p < .001, GHRM = Green HRM practices, GKS = Green knowledge sharing, GPTI = Green product innovation, GPSI = Green process innovation, GCA = Green competitive advantage, GHC = Green human capital.

Furthermore, Hypothesis 4a is supported by the fact that the impact of GPTI on 10.13039/100005839GCA (*β* = 0.294, *t* = 3.452, *p* < .01) was positive and highly significant (see Table 6). GPSI shows a substantial positive correlation with GCA, according to Hypothesis 4b. This hypothesis is supported by the fact that the impact of GPSI on 10.13039/100005839GCA (*β* = 0.395, *t* = 4.524, *p* < .01) was positive and highly significant as well (see Table 6).

The results indicated that all the direct relationships in the study were positive and highly correlated. This was the confirmation of all the direct hypotheses that were made in the study. Hence, the results were equal to as per the desired consequences as well as the results validate all the hypotheses.

### 4.4 Mediation analysis

According to Hypotheses 5a, 5b, 6a, and 6b, variables GKS, GPTI, and GPSI mediate the relationship between GHRM and GCA. The stated path analysis (see Table 6) revealed that GHRM has a particular indirect effect on GPTI (*β* = 0.249, *t* = 3.814, *p* < .01) from GKS given that these hypotheses meet the mediation conditions [89], according to the required path analysis in hypothesis 5a. So, this mediation **(**GHRM→GKS→GPTI) was also highly significant and positive. The necessary path analysis in hypothesis 5b shows that GHRM has a distinct indirect effect on GPSI through GKS (*β* = 0.255, t = 3.829, p < .01), according to hypothesis 5b.

According to the path analysis presented (see Table 6), GKS influences GCA indirectly (*β* = 0.183, *t* = 3.325, *p* < .01) from GPTI, according to the required path analysis in hypothesis 6a. So, this mediation **(**GKS→GPTI→GCA) was highly significant and positive as well. GKS is affected indirectly by GPSI on GCA (*β* = 0.252, *t* = 4.231, *p*.01), according to the required path analysis in hypothesis 6b. Hence, this mediation **(**GKS→GPSI→GCA) was also highly significant and positive. These results suggested that GKS, GPTI and GPSI were acting as partial mediators. As a result, hypotheses 5a, 5b, 6a and 6b were found to be validated.

### 4.5 Moderation analysis

Hypothesis 7 states that GHC positively and strongly moderates the relationships between GHRM and GKS. The path analysis results showed that GHC (*β* = 0.410, *t* = 5.430, *p* < .01) significantly contributed to GKS, and its moderation (GHRM × GHC) on GKS (*β* = 0.152, *t* = 2.873, *p* < .01) was positive, highly significant, and extremely strong, assisting and inferring that the relationship between GHRM and GKS strengthens with increasing levels of GHC.

Moderation was analyzed with simple slope analysis. The centered curve has without any effect of moderation, indicating GHC at mean. The analyses indicated that when the moderating effect (GHRM × GHC) was stronger (at +1 SD), the curve became steeper showing the vigorous positive impact on GKS (Fig. 4). Moreover, when the moderating effect (GHRM × GHC) was weaker (at −1 SD), the curve became flatter decreasing the impact on GKS. Hence, these analyses resulted that there is a highly significant positive moderation (GHRM × GHC→GKS) was present in the relationship, validating hypothesis 7.

## 5 Discussion

In complex and evolving settings, although, just gathering assets is lacking to hold a GCA. To adapt to a changing environment, resources must be quickly integrated and reconfigured into dynamic capabilities [90]. These dynamic capabilities lead to GCA and are in the form of Green HRM practices which was verified in the hypothesis 1. Information is also a significant key asset that has a high proclivity for producing GCA [91].

Enhancing staff efficiency [92] and determining innovative enactment, organizations should construct information-based unique abilities like information procurement, incorporation, and blend [93]. Information arranged experts would better energize Green HRM practices and GKS, by liking employees for insight, skills, familiarity, and commitments about GI during production. This was validated in hypothesis 2.

Manufacturing organizations should hire the staff with green recruitment and selection, training and development, performance appraisals, rewards and promotions, according to AMO theory. This all may be achieved by setting green goals for them also. Hence, cultivate a group philosophy that encourages the improvement of green knowledge-based unique capacities, bringing about better degrees of innovation achievement and, surprisingly, a GCA. Hence, this was validated in hypotheses 3a, 3b, 4a and 4b.

The PLS path model demonstrated the solid affirmative connection of variables, as anticipated. This study implies that in order to stimulate GKS during the social and job ranks, GHC should incorporate knowledge orientation with the help of Green HRM practices. This will increase colleagues’ ability to share knowledge and expertise with one another so that it may benefit to employees and organizations as well.

Performance appraisals and rewards help out to motivate the employees towards the green goals. In this way, employees may improve their contributions, competencies, service qualities and degrees of teamwork to protect the environment, in order to turn it to GHC. This strategy would foster GI and generate knowledge-based dynamic skills for achieving GCA in footings of organizational triumph, organizational job efficiency, and the whole show. Hence, the strong AMO framework application adds fuel to the NRBV framework according to this study. It also works on the firm's green status. Therefore, hypotheses 5a, 5b, 6a and 6b are accepted and verified the mediators.

This exploration's aim is a demonstration of GHC highly significant affects over GKS in SMMEs. This research found that this has a moderate positively and high significance impact on GKS, which supports hypothesis 7. Previous research, such as Ref. [94] has found that an organization's inside asset based human resources is a deciding component of complex performance in the organization. Moreover, this study found that assuming an organization's way of life is creative and solid, GHC advances toward increments and impacts organizational learning and information efficiency simultaneously. Because industry practices are constant across cities and countries, all SMMEs can be regarded similar in terms of working culture and management.

### 5.1 Theoretical contributions

The study contributed towards AMO theory and NRBV by uniting these in order to get GCA. Green HRM practices have added that organizations may set green goals for the employees and provide them the way out in the form of training and development [95]. This research adds to the knowledge that organizations may include green behaviors for performance appraisals, rewards and promotions [23]. This will motivate the employees in order to articulate GKS as an opportunity.

The main contribution of this study is to introduce the GHC as a moderator. This concept explains that if there are already available environment-friendly and skilled employees then they may share their green knowledge with newly recruited employees in order to gel as united workforce. This behavior may rush towards the GPTI and GPSI more aggressively rather with the absence of GHC. Hence, these GI dimensions lead to GCA according to NRBV [7].

The most significant contribution of this study is to join AMO framework with NRBV framework [95]. Organizations may implement this model in order to earn green repute in the customers and this may bring positive impact on the turnover [96]. Moreover, it may cut costs in order to make innovations about the product or process. This study provides the breakthrough in the form of novel concept of GCA [97]. Organizations may gather this advantage by putting this study's model into action. When employees begin to share the green information with one another then the result may lead to GI dimensions, which is a mediator of GCA in this study. Hence, this is the first study that provides Green HRM practices as the high-performance work practices [21] in order to implement GCA from the NRBV framework.

This investigation improves the green knowledge of the organizations that in order to develop the employees cost-efficient and innovative, these may give suggestions about the materials that produce the least amount of pollution or energy consumption [7]. Moreover, this study suggests to the organizations that the products contain the lowest amounts of resources by implementing the recycling process with their productions. This allows the organizations to develop the eco-friendly design of the products in order to achieve the green position with respect to competitors which may difficult to imitate.

### 5.2 Managerial Implications

A organization's GHC is the underlying leverage resource required to receive the possible rewards like GKS [98]. As a result of these findings, managers should focus more on developing a strong GKS culture and developing appropriate techniques to cultivate an organizational innovation climate about the environment that supports employee ideas, resulting in a 10.13039/100005839GCA. As a result, organizational GIs can assist businesses in meeting legislative criteria for environmental protection. It is additionally basic for SMMEs to recruit employees who are enthusiastic regarding environmental protection. Different checks may be created for this reason at the hour of recruiting. The current study proposes that executives ought to keep up with green discipline by rebuffing or refining employees who do not follow manufacturing environmental strategies.

The analysis recommends that investing in environmental management can help an organization earn a positive reputation among stakeholders, who are increasingly demanding and pressuring businesses to make strides toward environmental friendliness in every one of their cycles, products, and managements. The modern production to claim knowledge as the primary source of production processes, replacing land, labor, money, machinery, and other organization fixed assets [99]. AMO theory explains knowledge sharing as opportunity for the organization to affect modification. The concepts like high-performance work systems like Green HRM practices in AMO theory and product stewardship as well as pollution prevention of NRBV framework may help to achieve the technology enhancement in the organizations. Because this study only sampled from one geographic location in Pakistan, the findings may not be entirely generalizable in other geographical, social, and economic contexts. These findings, however, provide managers with important insights to solve their business and environmental challenges.

### 5.3 Limitations and future research directions

The study design was longitudinal time-lagging, and the specialist accepts that future examination ought to use a cross-sectional study to allow for more flexible investigation of mediating effects over a single time. Furthermore, the findings inspire academics to take a gander at other likely conditions, for example, the study suggested that future research take into account organizational characteristics like green strategy and transformational leadership, which could lead to some fascinating findings.

The study should probe deeper into the component of this relationship, as well as consider other captivating builds like environmental knowledge, investor pressure, favorable to natural qualities, and green organizational culture. Moreover, future research should consider the industrial sector as well to verify the results at the larger scale. The sample for this study was chosen from a particular region (Punjab province) in Pakistan; therefore, these findings may not be fully generalized to other local and international geographical, social, and economic contexts due to cultural and environmental variations. Future research should consider a more diverse and a larger sample to gain more generalizable outcomes. Similar studies can be conducted in western cultural environment including newly industrialized countries with environmental concerns, such as South Korea or Singapore. Future examination might also adopt a mixed-method approach to concentrate on the connection between the variables of this research model.

### 5.4 Conclusion

Green HRM practices predict GCA for SMMEs. This phenomenon is the result of a moderated mediation process. In a sequential development, the green HRM practices promote GKS that lead to green product and GPSIs, which in turn contribute to GCA. Green human capital plays the role of a moderating variable in the process. It interacts with the green HRM practices, enhances GKS, and thus boosts GCA by increasing green product and process innovations. These findings are well supported by the 10.13039/100006116AMO and NRBV theories and have significant relevance for the SMMEs seeking a green edge over their industrial competitors.

## Data availability statement

Data will be made available on request.

## CRediT authorship contribution statement

**Khurram Mustafa:** Writing – original draft, Investigation, Formal analysis, Conceptualization. **Md Billal Hossain:** Visualization, Funding acquisition, Methodology, Supervision. **Farooq Ahmad:** Methodology, Formal analysis, Conceptualization, Visualization. **Faisal Ejaz:** Data curation, Methodology, Writing - review & editing. **Hafiz Ghufran Ali Khan:** Validation, Data curation, Software, Visualization. **Anna Dunay:** Validation, Resources, Methodology, Funding acquisition, Conceptualization, Project administration.

## Declaration of competing interest

The authors declare that they have no known competing financial interests or personal relationships that could have appeared to influence the work reported in this paper.
