# Supplementary material for: Green human resource management practices to accomplish green competitive advantage: A moderated mediation model
Source: Heliyon. 2023 Oct 31;9(11):e21830. doi: 10.1016/j.heliyon.2023.e21830 (PMC10661352; doi:10.1016/j.heliyon.2023.e21830)
Supplement: Multimedia component 1 [file mmc1.docx]

# Green HRM Practices

## Reference

J. Dumont, J. Shen, X. Deng, Effects of Green HRM on Employee Workplace Green Behavior: The Role of Psychological Green Climate and Employee Green Values, Hum Resour Manage. 56 (2016) 613–627. https://doi.org/10.1002/hrm.21792.

## Items

1. My organization sets green goals for its employees.
2. My organization provides employees with green training to promote green values.
3. My organization provides employees with green training to develop employees’ knowledge and skills required for green management.
4. My organization considers employees’ workplace green behavior in performance appraisals.
5. My organization relates employees’ workplace green behaviors to rewards and compensation.
6. My organization considers employees’ workplace green behaviors in promotion.

# Green Knowledge Sharing

## Reference

H. Lin, Knowledge sharing and firm innovation capability : an empirical study, Int J Manpow. 28 (2007) 315–332. https://doi.org/10.1108/01437720710755272.

## Items

1. I always share green knowledge obtained from newspapers, magazines, journals, television and other sources.
2. I enjoy sharing environment-friendly knowledge with my colleagues.
3. In my organization, people share expertise from work experience with each other.
4. Sharing my knowledge with colleagues is pleasurable.
5. I believe that knowledge sharing can benefit all parties involved.

# Green Innovation

## 1. Green Product Innovation

## Reference

Y.S. Chen, S.B. Lai, C.T. Wen, The influence of green innovation performance on corporate advantage in Taiwan, J Bus Ethics. 67 (2006) 331–339. https://doi.org/10.1007/s10551-006-9025-5.

## Items

1. The organization chooses the materials of the product that produce the least amount of pollution for conducting the product development or design. The organization chooses the materials of the product that produce the least amount of pollution for conducting the product development or design.
2. The organization chooses the materials of the product that consume the least amount of energy and resources for conducting the product development or design.
3. The organization uses the fewest amounts of materials to comprise the product for conducting the product development or design.
4. The organization would circumspectly deliberate whether the product is easy to recycle, reuse, and decompose for conducting the product development or design.

## 2. Green Process Innovation

## Reference

Y.S. Chen, S.B. Lai, C.T. Wen, The influence of green innovation performance on corporate advantage in Taiwan, J Bus Ethics. 67 (2006) 331–339. https://doi.org/10.1007/s10551-006-9025-5.

## Items

1. The manufacturing process of the organization effectively reduces the emission of hazardous substances or waste.
2. The manufacturing process of the organization recycles waste and emission that allow them to be treated and re-used.
3. The manufacturing process of the organization reduces the consumption of water, electricity, coal, or oil.
4. The manufacturing process of the organization reduces the use of raw materials.

# Green Competitive Advantage

## Reference

Y.S. Chen, C.H. Chang, Enhance environmental commitments and green intangible assets toward green competitive advantages: An analysis of structural equation modeling (SEM), Qual Quant. 47 (2013) 529–543. https://doi.org/10.1007/s11135-011-9535-9.

## Items

1. The organization has the competitive advantage of low cost about environmental protection or green innovation compared to its major competitors.
2. The quality of the green products or services that the organization offers is better than that of its major competitors’ green products or services.
3. The organization is more capable of Research & Development about environmental protection or green innovation than its major Competitors.
4. The organization has better environmental managerial capabilities than its major competitors.
5. The organization’s environmental profitability is better.
6. The growth of the organization about green products or services exceeds that of its major competitors.
7. The organization is the first mover in some important fields about green products or services and occupies some important positions.
8. The environmental image of the organization is better than that of its major competitors.
9. The major competitors of the organization cannot imitate its green products or services easily.
10. The major competitors of the organization cannot imitate its environmental ideas easily.
11. The major competitors of the organization cannot replace its distinctive position about environmental manage mentor green innovation easily.

# Green Human Capital

## Reference

Y.S. Chen, The positive effect of green intellectual capital on competitive advantages of firms, J Bus Ethics. 77 (2008) 271–286. https://doi.org/10.1007/s10551-006-9349-1.

## Items

1. Whether the productivity and contribution of environmental protection of the employees in the organization is better than those of its major competitors.
2. Whether the employees’ competence of environmental protection in the organization is better than that of its major competitors.
3. Whether the product or service qualities of environmental protection provided by the employees of the organization are better than those of its major competitors.
4. Whether the cooperative degree of team work about environmental protection in the organization is more than that of its major competitors.
5. Whether the managers can fully support their employees to achieve their jobs of environmental protection.
